# Effectiveness of Photobiostimulation and Ablation With Diode Laser in Oral Squamous Papilloma: A Case Report

**DOI:** 10.1155/crid/6293919

**Published:** 2025-06-22

**Authors:** J. A. Costa, M. B. Alvarez, L. Monteiro, P. Rompante, F. Salazar, M. Relvas, M. I. Câmara

**Affiliations:** UNIPRO, Oral Pathology and Rehabilitation Research Unit, University Institute of Health Sciences (IUCS-CESPU), Gandra, Portugal

**Keywords:** common wart, diode laser, human papillomavirus lesion, oral squamous papilloma, photobiostimulation, squamous papilloma

## Abstract

This study evaluates the therapeutic effectiveness of diode laser and photobiostimulation (PBS) in the treatment of an oral squamous papilloma in the lingual mucosa in a 69-year-old male. The patient was subjected to complete hematological examination, and all parameters were within normal limits. The kind of damage, accessibility, aesthetics, and possibility of bleeding all played a role in the choice of therapy. The lesion was totally excised using the diode laser, and PBS was applied to contribute to healing and reduction of bleeding and inflammation. The patient had no pain throughout this process, which was shown to be a fast and efficient method of therapy. After 30 days, the lesion was fully healed, and 1 year of follow-up has been completed positively with no recurrences. In conclusion, PBS promoted rapid healing and diode laser is an alternative in these types of oral lesions and a good substitute for traditional surgery.

## 1. Introduction

Since their first development in 1960, lasers have been employed in almost all contexts of modern dentistry, such as gingival melanin hyperpigmentation corrections, nonsurgical treatment of periodontitis and peri-implant lesion, tooth decay removal, reinforcing restorations, and tooth bleaching. Other applications include lesions' treatment with minimal scarring, reduced bleeding, edema, and pain [1]. The most frequently used lasers in oral surgery are the CO_2_ laser, Erbium family lasers, Nd:YAG laser, and diode laser [2]. Diode laser has become very widespread in dentistry because it is easy to use for small soft tissue surgery. Due to its photothermal effect, diode laser is used for the removal of small lesions of the oral mucosa [3].

Benign oral lesions can be treated with laser therapy with success rates ranging between 64% and 100% [4]. Several studies demonstrated that laser therapy, namely diode laser, is an effective alternative to conventional surgery in the treatment of squamous papilloma (SP) [5].

Laser therapy can also be used to induce a photobiomodulatory effect on cells and tissues, contributing to the modulation of cell behaviors and enhancing the processes of tissue repair and regeneration. Photobiostimulation (PBS), also known as low-level laser therapy, can induce cell proliferation and enhance stem cell differentiation, being a noninvasive method that contributes to pain relief, reduces inflammation, and enhances healing and tissue repair processes [6–8].

SP is a common lesion and the most frequent benign oral epithelial entity in both children and adults [9]. Adults experience the highest incidence, with increases between the third and fifth decades. The palate and tongue are most commonly affected. Clinically, SP is characterized by exophytic projections as “finger-like,” usually pedunculated, with color ranging white to pink/red. “Cauliflower” or “warty” is also a common surface descriptor [10]. There are several methods to treat SP, such as laser ablation, conventional surgical excision, systemically used interferon, cryotherapy, and electrocauterization [11].

This study was aimed at evaluating the effectiveness of PBS and diode laser in the treatment of SP in the left lateral border of the lingual mucosa in a 69-year-old male.

## 2. Case Presentation

**Figure 1:**
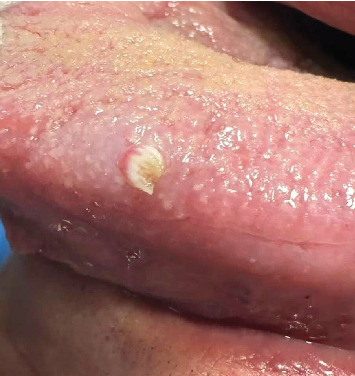
Clinical appearance of oral squamous papilloma.

A 69-year-old Caucasian male was referred to the Filinto Batista Universitary Clinic of the IUCS-CESPU, Portugal. The patient reported noticing an oral lesion for over 2 months, in the lateral border of the left side of the tongue. There was no history of any relevant systemic disease or habits. On oral examination, a white to pinkish lesion, measuring 5 mm, with a warty texture in the shape of a cauliflower was observed (Figure 1).

**Figure 2:**
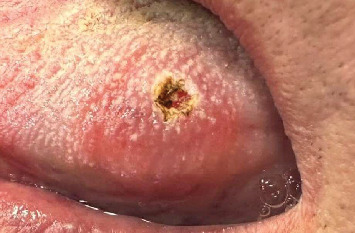
Clinical appearance after laser ablation.

Following clinical examination, the patient is informed that his lesion appears to correspond to a common wart or SP that shares similar characteristics. The patient was subjected to complete hematological examination, and all parameters were within normal limits. After infiltration of local anesthesia (articaine 4% 1:100,000, approximately 1 mL), the complete excision of the lesion (Figure 2) was performed with diode laser (Lasotronix Smart M Pro, Lasotronix Sp. Zo.o., Piaseczno, Poland), at wavelength 980 nm, with a power output of 10 W, which provides the fastest cutting and improved coagulation.

**Figure 3:**
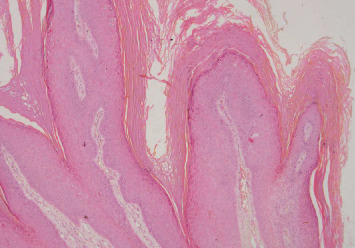
Histopathological examination showing filiform structures composed of connective papillae and lined by hyperkeratinized stratified epithelium.

After excision, PBS was applied on the treated area to accelerate tissue healing, reduce inflammation, provide analgesic action, and enhance the regenerative effect. In this case, our selection was a 635-nm wavelength, 100-mW power, and energy density of 3.0 J/cm^2^ for 15 s around the entire lesion extension. Histopathological assessment showed filiform structures composed of connective papillae and lined by hyperkeratinized stratified epithelium, confirming the diagnosis of SP (Figure 3). No signs of malignancy were present.

**Figure 4:**
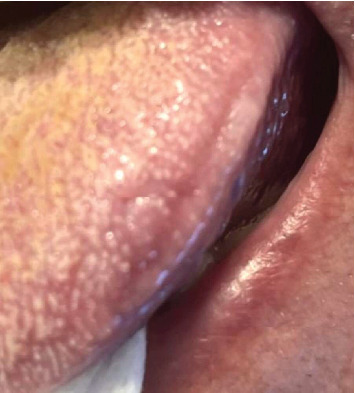
Clinical appearance after 1 month.

One week after the treatment, the patient described that no pain medication was needed and no eating or speech impairment. Two weeks later, the patient was evaluated, presenting good healing, no pain or discomfort (Figure 4).

Clinical follow-up was performed at 1 and 12 months with complete healing and no symptoms. Until the last follow-up, there were no signs of recurrence of the lesion or any other symptom reported by the patient.

## 3. Discussion

The benign lesions, common HPV infections of the oral cavity, comprise verruca vulgaris, SP, condyloma acuminatum, and multifocal epithelial hyperplasia, which share clinical and histologic features among themselves [9].

SP is a benign growth of the stratified squamous epithelium that has a sessile or pedunculated base and appears as a verrucous, papillary exophytic mass or cauliflower and generally measures less than 1 cm. Although any cavity surface may be impacted, the tongue and soft palate are the most often afflicted areas [12]. Abbey et al. observed that the palate had a higher frequency of lesions than other areas including the lips and lateral border of the tongue [10]. Carneiro et al. conducted a study in 12 individuals with suspected SP. The palate (33.3%), lip (16.7%), labial commissure (8.3%), and tongue (41.7%) were the most common sites [13].

Histopathologic criteria for SP include finger-like projections of the squamous epithelium, a normal maturation pattern, hyperparakeratosis in the epithelium, koilocytosis due to perinuclear cytoplasmic vacuolization of the epithelium's spinous layer cells, pyknosis, and the occasional presence of basilar hyperplasia [14].

The conditions condyloma acuminatum, verruca vulgaris, and papillary hyperplasia are included in the differential diagnosis of solitary type SP. Inflammatory papillary hyperplasia displays irritation as the clear causal factor [15]. Verruca vulgaris is a benign epidermal growth that often affects mucous membranes and epithelial tissues. Malignant changes are rare. It is frequently brought on by HPV-2 and HPV-4, and few cases are reported on the tongue. Little pink or white nodules, which can be sessile or pedunculated, usually with surface shape like a cauliflower are the hallmark of condyloma acuminatum [16]. They spread through the tongue, lips, palate, and floor of the mouth [17]. Condyloma acuminatum is larger than SP and are more frequently found in multiples [9]. Some research supports the combination of SP and condyloma acuminatum because both lesions are based on the histologic and clinical manifestations [16].

Smoking, concurrent infections, malnutrition, hormonal alterations, and immunological changes such as those found in persons with HIV, who typically have several oral lesions, have an impact on the development of these lesions [11].

The treatment of choice in this case for SP is the diode laser, whose benefits for the patient are the following: less time spent in the chair, better patient acceptance of the procedure, hemostasis, absence of postoperative swelling, increased patient compliance with less pain, shorter recovery times, no or less use of postoperative medications, safer operations on cardiac and coagulopathic patients, and antimicrobial effects [18]. Choosing the appropriate laser power and duration setting based on the tissue being treated is another crucial factor. Treatment-related complications include glandular stenosis, nerve injury, necrosis, and perforation of the mucosa covering the lesion. To prevent these complications, precautions should be made, such as avoiding the fibertip of the laser not being held in the same place for too long and cooling of the surface to avert ulceration [19]. Additional methods for treatment include electrocautery, cryosurgery, intralesional interferon injections, and surgical excision [20].

Endre Mester first described the benefits of PBS in the 1960s, and researchers from the National Aeronautics and Space Administration (NASA) employed it to improve healing in space. As PBS effects promote cell viability by promoting the creation of adenosine triphosphate (ATP) in mitochondria and cell membrane photoreceptors, laser and LED light are utilized to speed up healing [6]. PBS involves applying light, often from a low-power light source (laser or LED), to promote tissue healing, reduce inflammation, provide analgesia, improve bone remodeling and repair, immune system regulation, normalization of atypical hormonal activity, restoration of normal neuronal function after damage, and pain reduction [7].

About pain, it has been determined that laser therapy is superior at reducing discomfort and speeding up the healing process [21]. At the cellular level, it increases tissue oxygenation, improves microcirculation, stimulates the growth of mesenchymal cells, enhances the rate of tissue re-epithelization, and improves fibroblast/extracellular matrix proliferation. The benefits of laser therapy for pain relief are achieved by modulating the metabolism of lymphocytes, decreasing the production of inflammatory mediators, and modifying the conduction of the nociceptor impulse [22].

In this case, at the postsurgical consultation, the patient reported no pain or signs of inflammation and good healing.

PBS is not based on warmth and does not ablate, which sets it apart from other light-based therapies. The factors employed, such as the light source, wavelength, energy density, light pulse shape, and the length of the laser application, determine how effective PBS is in the target tissue [23]. Fundamental to PBS are managed dosages and light conditions. With wavelengths typically between 600 and 700 nm and 780–1100 nm and an irradiance or power density of 5 mW cm^−2^ to 5 W cm^−2^ for lasers or LEDs, PBS is the utilization of light in the red or near-infrared area [8].

The World Association for Laser Therapy (WALT) has prescribed and suggested a protocol regarding the lines and dosages since certain results obtained from the biostimulation effects clearly reveal that laser therapy is beneficial in regeneration [24].

Regarding recurrence, the first 3 months is the higher-risk period [25]. According to this case, there were no signs of recurrence over a 1-year follow-up.

## 4. Conclusions

As we demonstrate in this case, the use of a diode laser for SP treatment proved to be an alternative to conventional surgery. The many benefits it provided are a rapid, painless, and successful procedure with good hemostasis, no need for sutures, wound sterilization, faster regeneration, no recurrences, and no need for medication to manage discomfort both during and after the procedure. The characteristics and advantages that the laser offers in these treatments were improved by the application of PBS.

## Data Availability

All data related to the presented manuscript are included within the article.
